# Oral cavity and oropharyngeal carcinoma disparities in age and survival in Indigenous and non-Indigenous populations of Queensland

**DOI:** 10.1186/s12885-023-11002-1

**Published:** 2023-06-03

**Authors:** Abdulrahman Sakeen Alkandari, Jemmi Ching Ying Ho, Siu Wai Choi, Peter Thomson

**Affiliations:** 1 Ministry of Health, Kuwait City, Kuwait; 2 Department of Anaesthesiology, Faculty of Medicine, University of Hong Kong, Pok Fu Lam, Hong Kong; 3 Department of Orthopaedics & Traumatology, Faculty of Medicine, University of Hong Kong, Pok Fu Lam, Hong Kong; 4 College of Medicine & Dentistry, James Cook University, Douglas, Australia

**Keywords:** Oral cancer, Indigenous Australians, Squamous cell carcinoma, Cumulative survival, Aboriginal and Torres Strait Islander

## Abstract

**Objectives:**

To investigate the risk and prognosis of oral squamous cell carcinoma (SCC) between Indigenous and non-Indigenous populations of Queensland.

**Materials and methods:**

Retrospective analysis of data from the Queensland Cancer Registry (QCR) between the years 1982–2018. Main outcome measures were age at diagnosis and cumulative survival to compare the risk and prognosis of oral SCC between the populations.

**Results:**

9424 patients with self-declared ethnicity were identified with oral SCC from the QCR, with a male to female ratio of 2.56:1. Of these patients, 9132 were non-Indigenous (96.9%) and 292 Indigenous (3.1%). Indigenous people were significantly younger at diagnosis (mean (SD) age 54.3 (10.1) years), compared to 62.0 (12.1) years in non-Indigenous people. Mean survival in the full cohort was 4.3 years (SD: 5.6), with Indigenous people presenting a significant shorter mean survival of 2.0 years (SD: 3.5) when compared with 4.4 years (SD: 5.7) in non-Indigenous people (*p* < 0.001).

**Conclusions:**

Indigenous Australians are diagnosed at a significantly younger age and present with worse survival and poorer prognosis. Due to missing variables in the Queensland Cancer Registry, it is not possible in the current study to ascertain the scientific or social reasons behind these disparities.

**Clinical relevance:**

Results from this study can inform public policy and raise awareness in Queensland regarding disparity in oral cancer prognosis.

**Supplementary Information:**

The online version contains supplementary material available at 10.1186/s12885-023-11002-1.

## Introduction

Oral squamous cell carcinoma (SCC) is the 12^th^ most common cancer worldwide in men [1]. It has a male predilection and disproportionately affects those of lower socioeconomic status and who engage in certain habits, such as smoking, excessive alcohol intake, or use of chewable tobacco or snuff [2]. Oral squamous cell carcinoma is usually detected at the later stages due to late presentation and lack of awareness in those patients. The survival rate of oral SCC is abysmal at only 50% at 5 years when the tumour is detected at TNM stage III and IV [3].

The purpose of this study is to investigate and compare the risk and survival rate of oral SCC between Indigenous and non-Indigenous populations within the state of Queensland, Australia, with the hope that results can inform governments regarding the allocation of resources and provide awareness of the current challenges.

The relative chance of survival for 5 years in the Indigenous population after any cancer diagnosis is 54% [4]. Population data show that Indigenous populations were also 1.4 times more likely to die from cancer than their non-Indigenous counterparts [5]. These lower rates of survival may be due to Indigenous populations being socially disadvantaged, such as in education and employment and may not seek medical help until the very late stages of disease. They are also more likely to partake in higher risk habits such as smoking, have worse nutrition, lower levels of physical inactivity, and poorer access to health services due to living in remote areas [6].

Based on data collected between 2007–2014 [4], Indigenous populations had a 42% 5-year survival rate compared to non-Indigenous populations at 66%, for all types of cancer. Between the years 2009–2013, 414 cases of head and neck cancers, including SCC were diagnosed in the Indigenous population, and between the years 2011–2015, 188 Indigenous Australians died from head and neck cancer [4].

Although population data looking at risk of cancer in Indigenous populations do exist [5], the information is not up to date and specific information on oral SCC have not been published. Therefore, our study aims to utilise data obtained from the Queensland Cancer Registry to conduct a comprehensive analysis on the differences in health outcomes in relation to oral SCC, between the Indigenous Aboriginal and Torres Strait Islander populations and the non-Indigenous populations in Queensland where Indigenous peoples make up around 4.6% of the total population. We also aim to investigate the age at diagnosis of oral SCC in these two populations.

## Materials and methods

Approval to conduct this retrospective study was obtained from the James Cook University Human Research Ethics Committee (Ref. H8609) and further approval under the Public Health Act 2005 provided by Queensland Health. The Queensland Cancer Registry (QCR) was accessed for the period 1982 (when data were first compiled) to 2018 (most recent available data); the dataset was received as a de-identified, password protected spreadsheet and managed in an encrypted Microsoft Excel spreadsheet using the Australian Code for the Responsible Conduct of Research.

Following data retrieval, all statistical analyses were performed using SPSS Statistics software, Windows version 28.0.1 (IBM Corp.). The number of patients, age at diagnosis and survival in years in Indigenous and non-Indigenous groups were analyzed using descriptive analysis, and were reported as mean, standard deviation (SD) and percentages. For data that are normally distributed, statistical significances between groups will be confirmed using 2 sample t-tests, or one-way ANOVA if there are more than two groups. If data are not normally distributed, non-parametric tests (Mann–Whitney test) will be used to test for differences between groups. Cox regression analyses were used to investigate overall survival between groups, using number of deceased patients and years between diagnosis and death as dependent variables to calculate hazard ratios (HR) and 95% confidence intervals for different ethnicities and cancer sites.

## Results

Following data analysis, with the confidence interval chosen was 95% and a P value of (< 0.05), a total of 9424 patients with self-declared ethnicity, were identified with oral SCC, with 9132 non-Indigenous (96.9%) and 292 Indigenous (3.1%) patients. Table 1 summarizes descriptive analyses between Indigenous and non-Indigenous groups. The overall mean (SD) age at diagnosis in all patients was 61.7 (12.1). Indigenous people were significantly younger at diagnosis (mean (SD) age 54.3 (10.1) years), when compared to 62.0 (12.1) years in non-Indigenous people (*p* < 0.001).

**Table 1:** Patient demographics

	**No. of patients**	**Mean age at diagnosis (SD)**	**No. of deceased (%)**	**Mean survival in years from diagnosis to death (SD)**
**All patients**	9424	61.7 (12.1)	5189 (55.1%)	4.3 (5.6)
Males	6777 (71.91%)	60.8 (11.2)	3652 (53.9%)	4.1 (5.5)
Females	2647 (28.09%)	64.2 (13.7)	1537 (58.1%)	4.7 (6.0)
**All Indigenous**	292	54.3 (10.1)	183 (62.7%)	2.0 (3.5)
Male	216 (73.97%)	54.3 (10.3)	130 (60.2%)	1.7 (3.3)
Female	76 (26.03%)	54.4 (9.5)	53 (69.8%)	2.5 (4.1)
**All non-Indigenous**	9132	62.0 (12.1)	5006 (54.8%)	4.4 (5.7)
Male	6561 (71.85%)	61.0 (11.2)	3522 (53.7%)	4.2 (5.5)
Female	2571 (28.15%)	64.5 (13.7)	1484 (57.7%)	4.8 (6.0)

A total of 6777 males and 2647 females were identified from the database, with a male to female ratio of 2.56:1. Females have an overall higher mean (SD) age at diagnosis at 64.2 years (13.7) than that of males at 60.8 (11.2) years (*p* < 0.001). Females also have an overall higher mean survival upon diagnosis (4.7 years, SD: 6.0) when compared to males (4.1 years, SD: 5.5) (*p* < 0.001).

Among all patients, 5189 patients (55.1%) had passed away at the time of data collection. There was a higher percentage of deceased patients among the Indigenous (62.7%), compared to the non-Indigenous patients (54.8%).

**Fig. 1:**
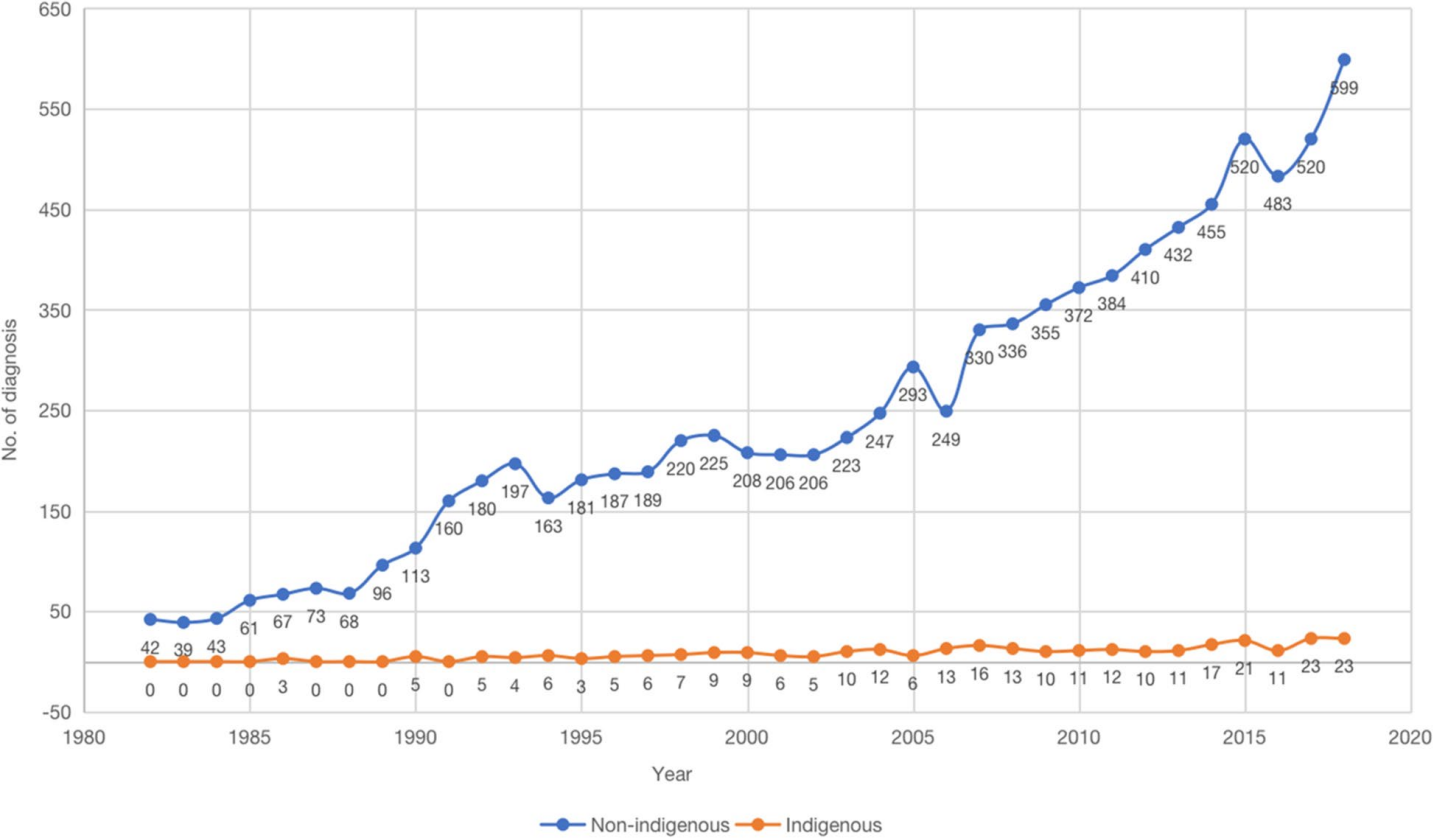
Trends in the number of diagnoses in Indigenous and non-Indigenous people from 1982 to 2018

**Fig. 2:**
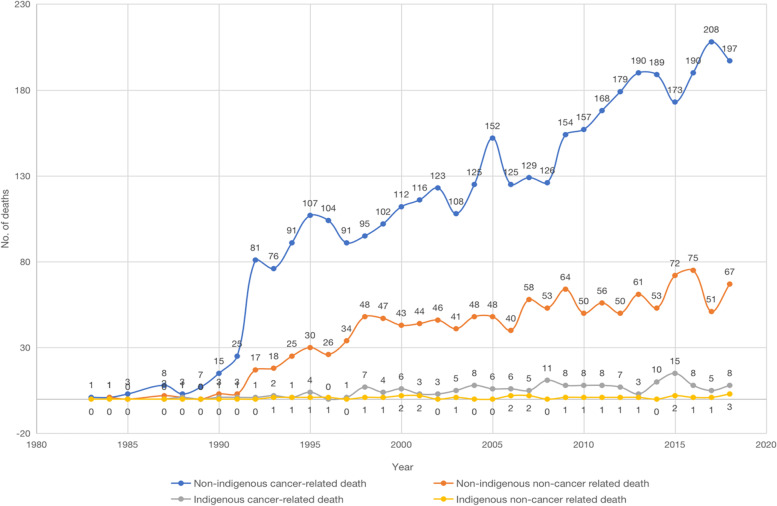
Trends in the number of deaths in Indigenous and non-Indigenous people from 1982 to 2018

Figure 1 shows the trends in the increasing number of diagnoses in Indigenous and non-Indigenous patients from 1982 to 2018. Both groups showed an increase over the 36-year period, with non-Indigenous people demonstrating a greater increase. As displayed in Fig. 2, trends in the number of deaths increased in both groups, where cancer-related deaths are more prevalent than non-cancer related deaths.

The mean survival time for patients was 4.3 years (SD: 5.6), with Indigenous people also presenting a significant shorter mean survival of 2.0 years (SD: 3.5) when compared with 4.4 years (SD: 5.7) in non-Indigenous people (*p* < 0.001). In terms of tumour site, Table 2 summarizes the mean age at diagnosis and mean survival in years between Indigenous and non-Indigenous people in various tumour locations. Significant differences in mean age at diagnosis and mean survival years between Indigenous and non-Indigenous people were observed in tumour sites recorded in the floor of mouth (*p* < 0.001), hard & soft palate (*p* < 0.001), tongue (*p* < 0.001) and tonsil (*p* < 0.001).

**Table 2:** Patient ethnicity, age at diagnosis and clinical outcome in corresponding tumour sites

	**No. of patients**	**Mean age at diagnosis (SD)**	***p*-value**	**No. of deceased (%)**	**Mean survival in years from diagnosis to death (SD)**	***p*-value**
**Tumour site (Total)**	9424	61.7 (12.1)		5189 (55.1%)	4.3 (5.6)	
Indigenous	292	54.3 (10.1)	< 0.001	183 (62.7%)	2.0 (3.5)	< 0.001
Non-Indigenous	9132	62.0 (12.1)		5006 (54.8%)	4.4 (5.7)	
**Oropharyngeal cancer**	2529	59.3 (10.6)		1139 (45.0%)	3.76 (5.1)	
Indigenous	90	52.6 (10.5)	0.948	49 (54.4%)	1.65 (3.0)	< 0.001
Non-Indigenous	2439	59.6 (10.5)		1090 (44.7%)	3.85 (5.2)	
**Oral cancer**	6756	62.6 (12.5)		2757 (40.8%)	4.47 (5.8)	
Indigenous	198	55.1 (9.8)	< 0.001	93 (47.0%)	1.9 (3.4)	< 0.001
Non-Indigenous	6558	62.8 (12.5)		2664 (40.6%)	4.6 (5.9)	
**Other cancers**	139	64.5 (12.0)		98 (70.5%)	3.7 (4.9)	
Indigenous	4	53.3 (11.6)	0.762	4 (100%)	0.0 (0.0)	0.004
Non-Indigenous	135	64.8 (11.9)		94 (69.6)	3.88 (4.9)	

Regarding tumour differentiation, the most common tumours were graded as moderately differentiated (47.84%), then poorly differentiated (24.9%), followed by well differentiated (11.18%) and undifferentiated (0.29%). Around 16% of tumours were not graded. Both Indigenous and non-Indigenous groups have the highest percentage of tumour differentiation graded as well differentiated.

**Fig. 3:**
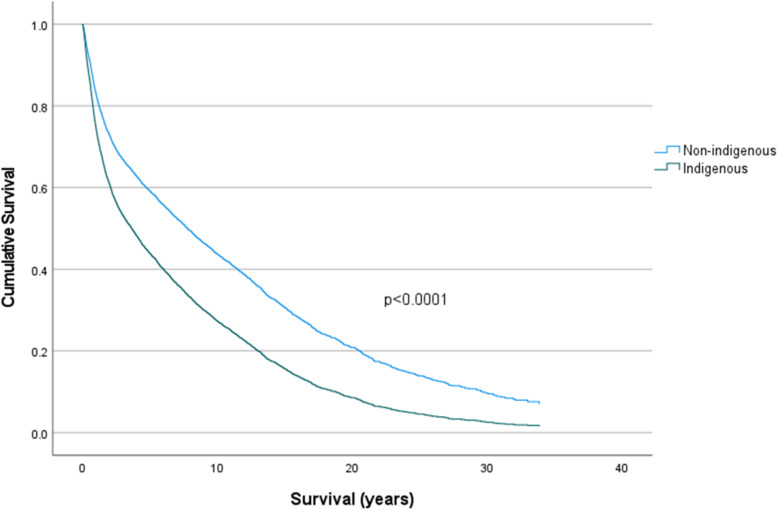
Cumulative survival in years between Indigenous and non-Indigenous people

Table 3 presents the hazard ratios in different ethnicities and tumour sites using Cox regression analysis. Applying non-Indigenous ethnicity as reference due to its higher mean survival in years, ethnicity exhibited a significant effect on mean survival with HR of 1.568 (95% CI: 1.352–1.817) and *p* < 0.001. Cumulative survival curves in Fig. 3 also indicates the impact of ethnicity on mean survival in years, which verified that Indigenous people had a lower mean survival and thereby a worse prognosis when compared to that in non-Indigenous people (*p* < 0.001). It is clear from Fig. 3 that around 50% of indigenous patients had passed away at five years post-diagnosis, compared to the non-indigenous patients, where around 50% of patients survived to around eight years post-diagnosis.

**Table 3:** Hazard ratios in Indigenous and non-Indigenous people among various tumour sites

	**Indigenous group**			**Non-Indigenous group**		
	Hazard ratio	95% Confidence interval	*p*-value	Hazard ratio	95% Confidence interval	*p*-value
**Ethnicity**	1.568	1.352–1.817	< 0.0001	Reference		< 0.0001
**Tumour site**						
**Buccal Mucosa and Vestibule**	1.764	0.393–7.912	0.459	1.601	1.377–1.861	< 0.0001
**Floor of Mouth**	1.338	0.475–3.770	0.582	1.475	1.337–1.627	< 0.0001
**Gingiva**	Reference		0.894	1.293	1.127–1.483	< 0.0001
**Hard & Soft Palate**	1.107	0.356–3.441	0.861	1.897	1.688–2.131	< 0.0001
**Labial Commissure**	-	-	-	1.267	0.699–2.299	0.435
**Mouth**	39.813	6.587–240.626	< 0.001	1.855	1.442–2.386	< 0.0001
**Oropharynx**	1.644	0.539–5.013	0.382	2.555	2.219–2.942	< 0.0001
**Retromolar**	2.102	0.523–8.456	0.295	1.695	1.462–1.966	< 0.0001
**Tongue**	1.161	0.424–3.185	0.771	1.262	1.167–1.365	< 0.0001
**Tonsil**	0.868	0.307–2.451	0.789	Reference		< 0.0001
**Overlapping lesion of lip, oral cavity and pharynx**	14.264	2.530–80.433	0.003	1.975	1.395–2.796	< 0.0001

For the analysis of overall survival data, the gingiva was used as a reference tumour site in Indigenous people; and tonsil was used as reference tumour site in non-Indigenous people to evaluate HR in various tumour locations. The highest HR was recorded in the mouth in Indigenous people, with HR of 39.18 (CI: 6.59–240.63) (*p* < 0.001); and oropharynx in non-Indigenous people with HR of 2.56 (CI: 2.22–2.94) (*p* < 0.001), indicating that these sites have a poorer prognosis when compared to other sites.

The remoteness profile of our sample of Indigenous peoples was ranked in descending order, outer regional 40%, major cities 22%, very remote 19%, inner regional 15%, not known 4%.

## Discussion

Oral health inequalities of Indigenous populations worldwide are similar in their inequality, even across very diverse geography, social structure, culture, and policy [7]. This inequality in oral health access, education, outcomes, and utilization would lead to lower attendance at oral healthcare clinics and fewer opportunities to spot oral malignancies at an earlier stage, resulting in under ascertainment and diagnosis of oral SCC in the Indigenous population. Studies on Indigenous populations have provided information on remote living, access to care, and access to follow-up treatment in case of disease.

Based on the number of patients diagnosed with oral SCC between 1982 and 2018 within our database, there is a clear disparity in survivorship between non-Indigenous and Indigenous populations. However, the rate of oral SCC diagnosis is similar in the two groups, the Indigenous population being 4% of the total population within the state of Queensland [8]. Our findings of decreased survival align with conclusions made in other studies which have shown an increased risk and worse prognosis in head and neck cancer, including oral SCC, as well as other types of cancers [4, 9]. Our data from Queensland does not indicate that the risk of oral SCC is higher in the Indigenous people, as the number of Indigenous patients in our database was only 3.09% of the total number of cases, but it is impossible to ascertain whether there is any under-reporting in remote-living Indigenous populations.

Our data showed that Indigenous patients were diagnosed at up to 10 years younger than their non-Indigenous counterparts. Epidemiological surveys have shown that Indigenous people were 2.9 times as likely to be current smokers compared to non-Indigenous populations, with those living remotely showing a higher trend (29% for major cities and 58% for very remote areas) [6]. In addition, Indigenous Australians aged 15 and over were 1.5 times as likely to be obese compared to non-Indigenous Australians [6]. Both smoking and obesity are independent risk factors for cancer development [10, 11]. Besides these lifestyle habits, Indigenous Australians also tended to live in remote communities [9, 12], therefore restricting their access to healthcare. This restricted access delays initial diagnosis and adds barriers to follow-up, demonstrated by the fact that Indigenous persons are 40% more likely to die from cancer [5] than a non-Indigenous person.

Risk of cancer death analysis (matched for age, sex and site) has shown that Indigenous populations tend to present at a later cancer stage to diagnosis than non-Indigenous populations [9]. Indigenous populations were also more likely to have distant metastasis at diagnosis when compared to non-Indigenous (31.3% vs 22.0,) [9, 12]. After adjusting for stage, remote-living Indigenous residents were at higher risk of cancer death than non-Indigenous residents of metropolitan areas [9]. Travelling a long distance to the hospital may also affect the provision of treatment, especially when it comes to the provision of radiotherapy, as well as during clinical follow-up post treatment. This underlines the importance of access to care that is trusted, timely, efficient, and provides a high quality of multidisciplinary treatment to a wide range of people. The lack of compliance to care and follow-up may also contribute to recurrence cases which have not been lodged in the Registry, second primaries, or if having undergone radiotherapy, radiation induced sarcoma.

Many cases of tonsillar cancer are caused by Human Papilloma Virus (HPV) infection, but as that data is not available in the QCR we are unable to correlate if the tonsillar cancer present in our data is due to HPV. However it is noted that the ratio of tonsillar cancer within our dataset is 23% for both the Indigenous and non-Indigenous population, and that the age of diagnosis and post diagnosis survival time is similar in tonsillar cancer to other cancer sites diagnosed and marked within the database.

It is noted that health insurance providers in Australia as well as state Patient Travel Subsidy Schemes do provide flight transportation from rural communities to the closest district hospital as well as hotel stay for one night for the diagnosis and treatment of cancer, though the approval of said schemes does take up to five working days prior to approval [13].

Improvement of trust within the Indigenous population in the healthcare system is necessary before any improvement in treatment could be achieved [14]. There is a stark difference in mean survival in years after diagnosis between Indigenous and non-Indigenous populations in Queensland. Contrary to expectations for the general population, although the Indigenous people present at a younger age, and would more likely have a reduced risk of comorbid disease, the survival rate is still lower than the older-aged, non-Indigenous patients. It is known that comorbidities can affect the prognosis and treatment provided [15], and reduce tolerance to treatment [15], so it would be interesting to investigate whether these patients did indeed suffer from a host of co-morbidities, contributing to worse prognosis.

The overweight/obesity rate is high in the Indigenous populations (80% at age > 35, 6], as is the rate of smoking, with 40.5% of the Indigenous population reporting as daily smokers (2018–2019) [6], compared to 11% for the Australian national average [16]. The shorter survival time and higher percentage of deceased in the Indigenous population studied in this current study could be explained by the unequal access to care, lack of trust of healthcare professionals, cultural differences, or difference in stage at presentation between the two groups. It is noted that in some instances the primary language of Indigenous populations may not be English, and as such their understanding of the treatment options offered, or their acceptance of it may be reduced [8].

Indigenous populations tend to live in very remote communities [12], with 32% of those who are classified as living in remote or very remote places being Aboriginal and Torres Strait Islander people based on estimated Indigenous population projections for 2021 [17]; this is reflected in our own Indigenous population data on remoteness. Living in remote areas can have a negative effect on follow-up and the treatment of recurrence, second primary diagnoses, or radiation induced sarcomas if the patient had undergone radiation treatment, as well as the management of sequelae from either the surgical or radiation treatment. This could be one of the reasons why the Indigenous population is diagnosed at a younger age in our study, as older patients when suspecting a lesion, may choose not to make the trip needed for diagnosis. It is noted that health insurance providers in Australia as well as state Patient Travel Subsidy Schemes do provide flight transportation from rural communities to the closest district hospital as well as hotel stay for one night for the diagnosis and treatment of cancer, though the approval of said schemes does take up to five working days prior to approval [13].

It is noted that in Australia there are healthcare institutions dedicated to *Aboriginal and Torres Strait Islander* (Indigenous) populations, National Aboriginal Community Controlled Health Organisation (NACCHO), and there are 28 such institutions within the state of Queensland, which are board elected and not state controlled [18]. However, the majority of these institutions provide primary care and outreach programs, whereas oral SCC is primarily treated in a surgical manner requiring specialist equipment and adequately trained personnel, which are usually located in large regional hospitals.

In addition to provision of better healthcare services, within the Indigenous population, cancer is considered a taboo subject, and is usually kept a secret, even from friends/family, and many may ignore symptoms as they view the diagnosis as a death sentence and do not see value in seeking treatment [14]. Those that do share their diagnosis with others may also face rejection, isolation, and fear, as there are some in the community that believe cancer is a contagious disease and may be transferred from one person to another [19]. This lack of support from those closest to the patient can lead to poor survivorship and the act of “giving up” [19]. Cultural beliefs of Indigenous populations also view the body as sacred, and that surgery is a violation of this sacredness [19], so patients may reject treatment on this basis. The combination of cultural practices, remote living and difficulty accessing healthcare may explain why the patients in our study presented at a younger age within the Indigenous populations, as the younger generation tend to be better educated and are more likely to seek treatment.

Our results show that the Indigenous population were found to have a worse prognosis when compared to non-Indigenous populations when the oral SCC was found to be at the floor of mouth, hard and soft palate, tongue, and tonsil. Assessment of these and other subgroups should be taken with caution as the number of Indigenous patients in each subgroup is very small. Tumours appearing in particular sites may be more prone to a lower survival rate, due to the stage of presentation or time till diagnosis; the patient may defer seeking help if it is painless and in an area that does not interfere with functions such as eating, speech, or swallowing.

It is noted here that the Queensland Cancer Registry does not collect data on cancer or TNM staging, a very important prognostic factor that is necessary to guide treatment modality. Therefore, we are unable to ascertain whether Indigenous patients were actually diagnosed at a later stage. Another failure of the registry is the designation of the anatomical site code of “mouth” for oral cancer, as “mouth” is all encompassing and does not provide accurate information as to the exact site of oral SCC. Risk and lifestyle factors were also not documented, such as smoking habit, alcohol intake, chewable tobacco use, recreational drug use, housing status, etc., so we were not able to conduct a more detailed analysis on the risk factors associated with oral SCC in Queensland.

### Limitations to this study

Although it is a legal requirement in Queensland for healthcare providers to lodge each cancer case in the QCR, there is no information regarding loss to follow-up nor any information on any cancer-related deaths which were not previously lodged as a diagnosed cancer case undergoing treatment or palliative care. In addition, patient compliance is not known to the QCR, as the requirement on the clinician is only to report the cancer and/or cancer death. There are no requirements to report follow-up compliance nor to report what treatments were given.

## Conclusion

This study has shown that when compared to their non-Indigenous counterparts, Indigenous Australians are diagnosed at a significantly younger age and present with worse survival and poorer prognosis. Due to missing variables in the Queensland Cancer Registry, it is not possible in the current study to ascertain the scientific or social reasons behind these disparities between populations.

It is suggested here that since Queensland has a system to systematically collect data on cancer cases, it would serve the community to add variables including cancer staging, treatment modalities and lifestyle habits of each patient. It would also be prudent to include attempts for follow-up, at least by telephone, and the success/failure of those attempts to be noted in the QCR to guide future policy.

Based on the data available and the likely under-representation of diagnosis in the Indigenous population in Queensland, oral health and oral cancer education, and oral screening in the Indigenous population could be a cost effective method in reducing the disease burden. This could be achieved in tandem with other screenings, such as for heart disease, diabetes mellitus, and lung, breast, colorectal, and prostate cancers. With regard to screening for oral malignancies, this should be conducted in primary care clinics in addition to outreach programmes accessible to the Indigenous populations in their own communities so that not only the “worried-well” are screened.

## Supplementary Information

**Additional file 1: Table 3.** Tumour differentiation in various cancer locations. **Supplementary Figure 1.** Cumulative survival in various reported tumour sites in Indigenous people. **Supplementary Figure 2.** Cumulative survival in various reported tumour sites in non-Indigenous people.

## Data Availability

We the authors submit that the raw data used for the analysis in this study is available upon request in writing to the corresponding author.
