# Supplementary material for: Oral cavity and oropharyngeal carcinoma disparities in age and survival in Indigenous and non-Indigenous populations of Queensland
Source: BMC Cancer. 2023 Jun 3;23:503. doi: 10.1186/s12885-023-11002-1 (PMC10239569; doi:10.1186/s12885-023-11002-1)
Supplement: Supplementary file 1 — Additional file 1: Table 3. Tumour differentiation in various cancer locations. Supplementary Figure 1. Cumulative survival in various reported tumour sites in Indigenous people. Supplementary Figure 2. Cumulative survival in various reported tumour sites in non-Indigenous people. [file 12885_2023_11002_MOESM1_ESM.docx]

Supplementary Data

Table 3. Tumour differentiation in various cancer locations

|  | | | **Total** | **Well differentiated** | **Moderately differentiated** | **Poorly differentiated** | **Undifferentiated** | **Unknown** |
| --- | --- | --- | --- | --- | --- | --- | --- | --- |
| **All patients** | | | 9424 | 1054 (11.18%) | 4508 (47.84%) | 2354 (24.9%) | 27 (0.29%) | 1481 (15.71%) |
|  | IndigenousIndigenous | | 292 | 25` (8.56%) | 116 (39.73%) | 82 (28.08%) | 1 (0.34%) | 68 (23.29%) |
|  | Non-Indigenous | | 9132 | 1029 (11.27%) | 4392 (48.09%) | 2272 (24.88%) | 26 (0.28%) | 1413 (15.47%) |
| **No. of deceased** | | | 5189 | 530 (10.21%) | 2694 (51.92%) | 1201 (23.15%) | 17 (0.33%) | 747 (14.40%) |
|  | | IndigenousIndigenous | 183 | 13 (7.10%) | 79 (43.17%) | 49 (26.78%) | 0 (0.0%) | 42 (22.95%) |
|  |  | Non-Indigenous | 5006 | 517 (10.33%) | 2615 (52.24%) | 1152 (23.01%) | 17 (0.34%) | 705 (14.08%) |
| **Mean age at diagnosis (SD)** | | | 61.74 (12.08) | 63.61 (13.16) | 61.73 (12.10) | 60.16 (11.33) | 57.93 (14.47) | 63.00 (11.97) |
|  | IndigenousIndigenous (SD) | | 54.31 (10.09) | 56.16 (10.36) | 53.93 (10.11) | 54.90 (9.26) | 43 (0.0) | 53.72 (10.97) |
|  | Non-Indigenous (SD) | | 61.97 (12.06) | 63.79 (13.17) | 61.94 (12.08) | 60.35 (11.35) | 58.50 (14.44) | 63.45 (11.84) |
| **Mean survival in years from diagnosis to death (SD)** | | | 4.27 (5.65) | 5.28 (6.35) | 4.67 (5.80) | 3.67 (5.27) | 4.53 (6.98) | 3.12 (4.81) |
|  | IndigenousIndigenous (SD) | | 1.95 (3.54) | 1.85 (3.96) | 2.42 (3.91) | 1.33 (2.79) | 0.0 (0.0) | 1.81 (3.45) |
|  | Non-Indigenous (SD) | | 4.36 (5.69) | 5.36 (6.38) | 4.74 (5.83) | 3.77 (5.32) | 4.53 (6.98) | 3.19 (4.87) |
| **Tumour site** | | |  | | | | | |
| **Buccal Mucosa and Vestibule** | | | 340 | 72 (21.18%) | 172 (50.59%) | 49 (14.41%) | 0 (0.0%) | 47 (13.82%) |
|  | IndigenousIndigenous | | 5 | 1 (20.00%) | 3 (60.00%) | 1 (20.00%) | 0 (0.0%) | 0 (0.0%) |
|  | Non-Indigenous | | 335 | 71 (21.19%) | 169 (50.45%) | 48 (14.32%) | 0 (0.0%) | 47 (14.03%) |
| **Floor of Mouth** | | | 1079 | 144 (13.34%) | 638 (59.13%) | 182 (16.87%) | 1 (0.09%) | 114 (10.57%) |
|  | IndigenousIndigenous | | 45 | 3 (6.67%) | 21 (46.67%) | 13 (28.89%) | 1 (2.22%) | 7 (15.56%) |
|  | Non-Indigenous | | 1034 | 141 (13.64%) | 617 (59.67%) | 169 (16.34%) | 0 (0.0%) | 107 (10.35%) |
| **Gingiva** | | | 519 | 99 (19.08%) | 292 (56.26%) | 82 (15.79%) | 2 (0.39%) | 44 (8.48%) |
|  | IndigenousIndigenous | | 5 | 0 (0.0%) | 3 (60.0%) | 2 (40.0%) | 0 (0.0%) | 0 (0.0%) |
|  | Non-Indigenous | | 514 | 99 (19.26%) | 289 (56.23%) | 80 (15.56%) | 2 (0.39%) | 44 (8.56%) |
| **Hard & Soft Palate** | | | 563 | 69 (12.26%) | 304 (53.99%) | 103 (18.29%) | 1 (0.18%) | 86 (15.28%) |
|  | IndigenousIndigenous | | 20 | 0 (0.0%) | 10 (50.00%) | 6 (30.00%) | 0 (0.0%) | 4 (20.00%) |
|  | Non-Indigenous | | 543 | 69 (12.71%) | 294 (54.14%) | 97 (17.86%) | 1 (0.18%) | 82 (15.10%) |
| **Labial Commissure** | | | 16 | 5 (31.25%) | 5 (31.25%) | 3 (18.75%) | 0 (0.0%) | 3 (18.75%) |
|  | IndigenousIndigenous | | 1 | 0 (0.0%) | 0 (0.0%) | 0 (0.0%) | 0 (0.0%) | 1 (100.0%) |
|  | Non-Indigenous | | 15 | 5 (33.34%) | 5 (33.34%) | 3 (20.00%) | 0 (0.0%) | 2 (13.33%) |
| **Mouth** | | | 86 | 13 (15.12%) | 31 (36.05%) | 15 (17.44%) | 1 (1.16%) | 26 (30.23%) |
|  | IndigenousIndigenous | | 2 | 0 (0.0%) | 0 (0.0%) | 0 (0.0%) | 0 (0.0%) | 2 (100.0%) |
|  | Non-Indigenous | | 84 | 13 (15.48%) | 31 (36.90%) | 15 (17.86%) | 1 (1.19%) | 24 (28.57%) |
| **Oropharynx** | | | 357 | 13 (3.64%) | 162 (45.38%) | 93 (26.05%) | 0 (0.0%) | 89 (24.92%) |
|  | IndigenousIndigenous | | 21 | 1 (4.76%) | 8 (38.10%) | 9 (42.86%) | 0 (0.0%) | 3 (14.29%) |
|  | Non-Indigenous | | 336 | 12 (3.57%) | 154 (45.83%) | 84 (25.00%) | 0 (0.0%) | 86 (25.60%) |
| **Retromolar** | | | 331 | 34 (10.27%) | 195 (58.91%) | 68 (20.54%) | 0 (0.0%) | 34 (10.27%) |
|  | IndigenousIndigenous | | 5 | 1 (20.00%) | 2 (40.00%) | 0 (0.0%) | 0 (0.0%) | 2 (40.00%) |
|  | Non-Indigenous | | 326 | 33 (10.12%) | 193 (59.20%) | 68 (20.86%) | 0 (0.0%) | 32 (9.82%) |
| **Tongue** | | | 3908 | 503 (12.87%) | 1860 (47.59) | 940 (24.05%) | 9 (0.23%) | 596 (15.25%) |
|  | IndigenousIndigenous | | 117 | 14 (11.97%) | 49 (41.88) | 27 (23.80%) | 0 (0.0%) | 27 (23.07%) |
|  | Non-Indigenous | | 3791 | 489 (12.90%) | 1811 (47.77) | 913 (24.08%) | 9 (0.24%) | 569 (15.01%) |
| **Tonsil** | | | 2172 | 99 (4.56%) | 824 (37.93) | 804 (37.02%) | 13 (0.59%) | 432 (19.89%) |
|  | IndigenousIndigenous | | 69 | 5 (7.25%) | 19 (27.54) | 24 (34.78%) | 0 (0.0%) | 21 (30.43%) |
|  | Non-Indigenous | | 2103 | 94 (4.47%) | 805 (38.28) | 780 (37.09%) | 13 (0.62%) | 411 (19.54%) |
| **Overlapping lesion of lip, oral cavity and pharynx** | | | 53 | 3 (5.66%) | 25 (47.17%) | 15 (28.30%) | 0 (0.0%) | 10 (18.87%) |
|  | IndigenousIndigenous | | 2 | 0 (0.0%) | 1 (50.0%) | 0 (0.0%) | 0 (0.0%) | 1 (50.0%) |
|  | Non-Indigenous | | 51 | 3 (5.88%) | 24 (47.06%) | 15 (29.41%) | 0 (0.0%) | 9 (17.65%) |

Supplementary Figure 1. Cumulative survival in various reported tumour sites in Indigenous people.


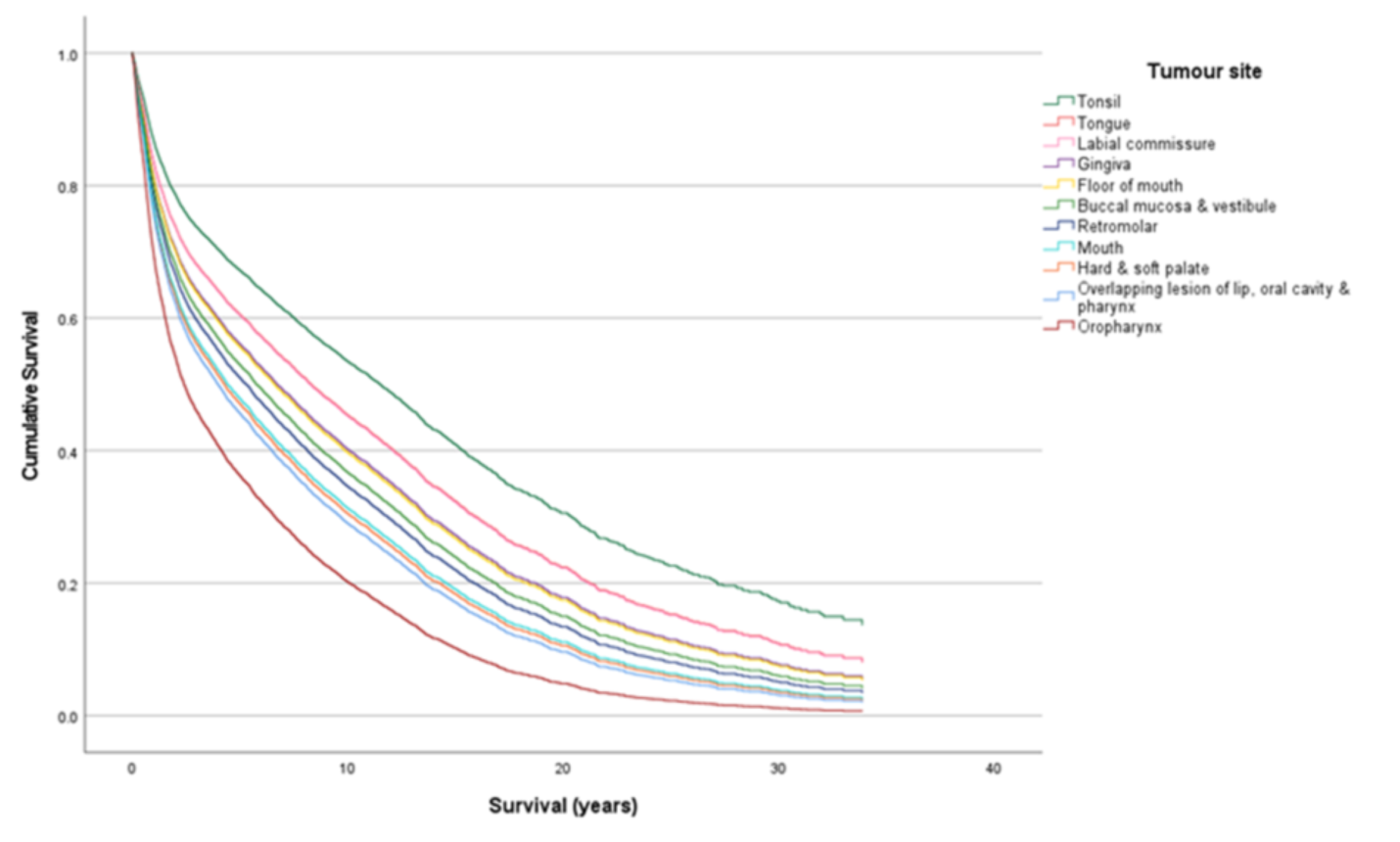


Supplementary Figure 2. Cumulative survival in various reported tumour sites in non-Indigenous people.


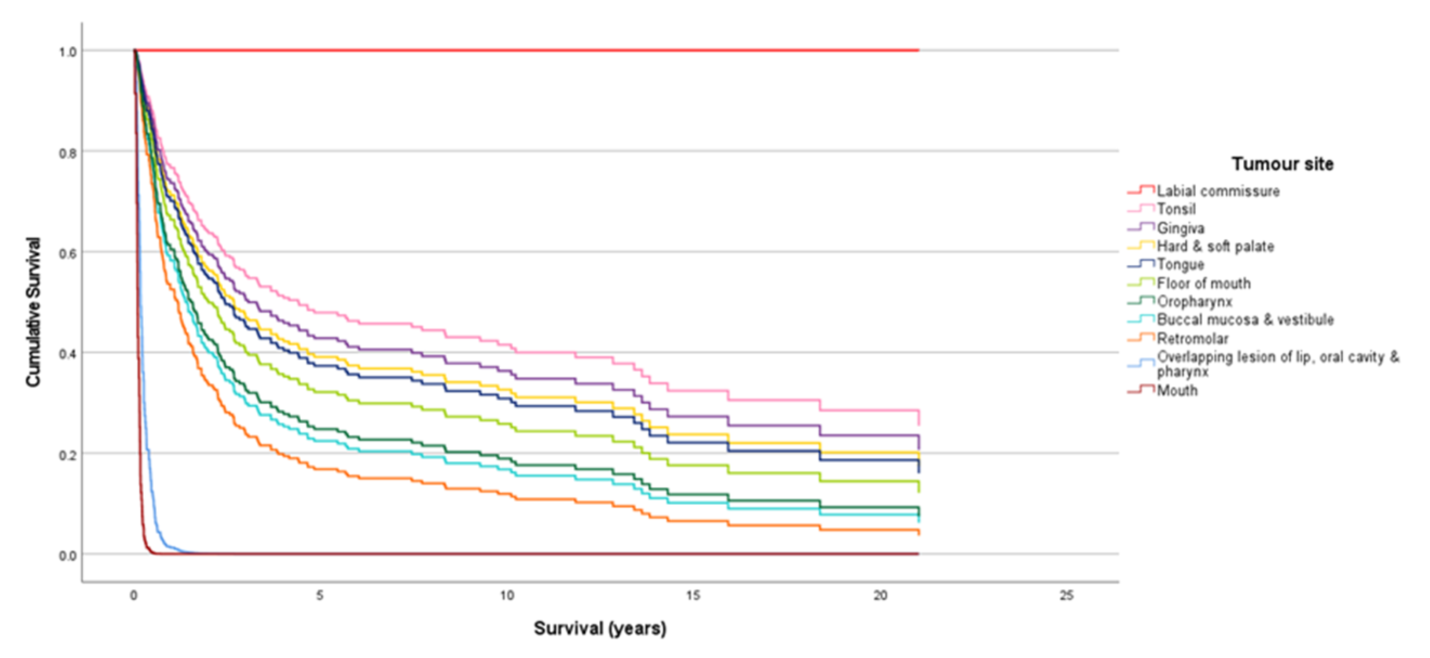


Figure legend:

Supplementary Figure 1. Cumulative survival in various reported tumour sites in Indigenous people.

Supplementary Figure 2. Cumulative survival in various reported tumour sites in non-Indigenous people.
